# The Economic Burden of Herpes Zoster in Individuals Aged 50 Years or Older and Those With Underlying Conditions in Italy

**DOI:** 10.1093/ofid/ofae738

**Published:** 2024-12-20

**Authors:** Eleftherios Zarkadoulas, Simona Comparoni, Riccardo Freguja, Roberto Santacroce, Melania Dovizio, Chiara Veronesi, Luca Degli Esposti, Ivan Gentile, Paolo Bonanni, Alessandro Rossi

**Affiliations:** GSK, Wavre, Belgium; GSK, Verona, Italy; GSK, Verona, Italy; GSK, Verona, Italy; CliCon S.r.l. Società Benefit Health, Economics & Outcomes Research, Bologna, Italy; CliCon S.r.l. Società Benefit Health, Economics & Outcomes Research, Bologna, Italy; CliCon S.r.l. Società Benefit Health, Economics & Outcomes Research, Bologna, Italy; Department of Clinical Medicine and Surgery, University of Naples Federico II, Naples, Italy; Università degli Studi di Firenze, Florence, Italy; Italian College of General Practitioners and Primary Care, Florence, Italy

**Keywords:** economic impact, health care resource utilization, herpes zoster, Italy, post-herpetic neuralgia

## Abstract

**Background:**

Risk of herpes zoster (HZ) infection increases with age and immunosuppression. We estimated the impact of HZ and post-herpetic neuralgia (PHN) on direct costs and health care resource utilization (HCRU) in patients ≥50 years, including those with comorbidities, as limited information exists in Italy.

**Methods:**

This retrospective analysis used reimbursement data from local health authorities in Italy (January 2009–June 2022). Cases of HZ and PHN identified by International Classification of Diseases, Ninth Revision, Clinical Modification codes and drug prescriptions were characterized and followed up for 1 year before and after the index date. The direct HCRU costs for patients with HZ/PHN were compared with those for patients without HZ/PHN.

**Results:**

Of the total 193 259 patients with HZ/PHN identified (mean age, 61.6 years), 145 923 were ≥50 years old (immunocompromised: 29.9%; ≥1 chronic condition: 76.1%). During follow-up, 18.8% of patients ≥50 years of age with HZ progressed to PHN complications, and 3618 hospital admissions were reported (median length of stay, 9 days). Drug prescriptions and all-cause hospitalizations were the main contributors to total annual direct health care costs, estimated at M€272 for patients with HZ/PHN, whose burden increased with age. Higher health care costs were observed in patients with HZ/PHN vs patients without HZ/PHN. Moreover, average health care costs were up to 4× higher for patients with HZ and PHN compared with those without PHN.

**Conclusions:**

HZ causes a significant economic impact on the health care system, driven mainly by high costs of medications and hospitalizations among older adults and those with comorbidities, particularly when complicated by PHN.

## Teaser

HZ causes significant economic impact on the health care system, driven mainly by high costs of medications and hospitalizations. As individuals age, this shows a sharp upward trend, particularly in those with comorbidities when complicated by PHN.

## Graphical Abstract

**Graphical abstract:**
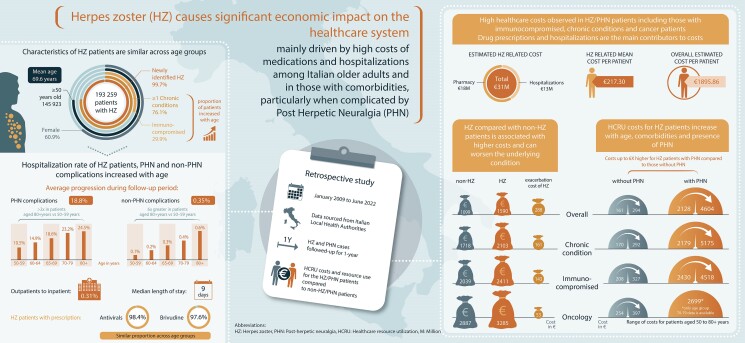

Herpes zoster (HZ) is a neurocutaneous and debilitating illness affecting millions of individuals worldwide. It is caused by reactivation of varicella zoster virus (VZV) that has remained latent in the cranial and dorsal root ganglia [1]. Reactivation is the result of the acceleration of natural immune senescence on account of aging or immunosuppression [2, 3]. Generally, it presents as a painful self-limited dermatomal rash, usually affecting 1 side of the body. About 30% of patients experience various complications, which may delay full recovery and thus have a negative impact on patients’ quality of life. One of the most common sequelae is post-herpetic neuralgia (PHN), a chronic neuropathic pain that can persist for months or even years after the disease has resolved [4, 5].

In their lifetime, nearly 30% individuals are at risk of acquiring HZ; the odds increase abruptly after 50 years of age, reaching 50% at 85 years of age [4]. In addition to age, patients with chronic comorbidities, like asthma and chronic obstructive pulmonary disease (COPD), are at increased risk of developing HZ compared with those without any such condition [6]. For example, patients with COPD are at 41% increased risk of HZ compared with healthy individuals. They are also more likely to experience an increase in episodes of COPD exacerbations around the time of HZ [7, 8]. Further, those who are immunocompromised (IC) because of an underlying disease (eg, malignancy) and on immunosuppressive therapy are also at an elevated risk of developing HZ [7, 9, 10].

Globally, due to changing demographics of older people, it is expected that the number of HZ cases will continue to increase. In Europe, individuals aged ≥65 years constitute the fastest growing age group at 19%, followed by North America (17%), Oceania (13%), and Asia (10%) [11]. Among European countries, Italy is leading, with the largest proportion of individuals aged >60 years (29.4%), followed by Germany (28.0%) [12]. Also, in Italy, 96.2% and 99.0% of the adult population by the age of 40 and 65 years, respectively, are at risk of developing HZ [13]. The overall incidence is estimated at 6.46/1000 person-years, increasing with age from 3.51 in the 50–54-years age group to 7.11 in those aged ≥80 years. Similarly, the overall proportion of PHN is 10.23% and increases with age [14].

The clinical and economic burden of HZ and PHN on societies and health care systems is significant [15]. In Italy, a study showed that HZ and PHN were associated with €41.2 million in annual costs (direct cost: €28.2 million; indirect costs: €13.0 million). However, this study used data from 2005 and earlier, focusing primarily on immunocompetent patients aged ≥50 years [16]. There are limited data on recent estimates, and with the growing proportion of older adults, the public health burden of HZ in the population is likely to increase in the coming years. Thus, there is a need to evaluate the impact of HZ and its complications, such as PHN, on health care resource utilization (HCRU) and costs. The primary objective of this study was to analyze HCRU and direct costs associated with outpatient and inpatient management of patients with HZ or/and PHN (>50 years) stratified by age group. The secondary objectives were to (i) compare the HCRU and costs for patients with HZ vs patients without HZ, (ii) estimate the proportion of HZ cases that progress to PHN or non-PHN complications, (iii) estimate the proportion of patients who progressed to an inpatient setting, including length of hospital stay, and (iv) describe demographic and clinical characteristics, including treatment patterns for HZ and/or PHN, stratified by age group.

## METHODS

### Study Design

The study was retrospective (Supplementary Figure 1). Data for this analysis were sourced from administrative databases housed at the Italian Local Health Authorities (LHA) covering ∼20% of the entire population from January 2009 to June 2022. The study consisted of the characterization and follow-up periods, each of 1 year's duration. During the characterization period, which was immediately before the index date (defined as the first day of HZ/PHN hospitalization at any level or an HZ-specific drug prescription), clinical features of patients were evaluated. For the 1-year follow-up period, HCRU and costs associated with patients included in the study were measured. For comparison, a control group was included, for which the index date was the first day of any hospitalization (inpatient) or any drug prescription (outpatient) during the inclusion year. These patients were followed for the same period of time as patients with HZ/PHN.

### Study Population and Data Sources

Details about the study populations and data sources used are presented in the Supplementary Data. International Classification of Diseases, Ninth Revision, Clinical Modification (ICD-9-CM) diagnosis codes were used to identify patients with HZ (Supplementary Table 1).

### Analysis

Data were analyzed using STATA SE, version 17.0 (Stata Corp LLC, College Station, TX, USA). Mean and SD were reported for continuous data, while frequencies and proportions were used for categorical variables.

Demographic and baseline clinical characteristics were summarized using descriptive statistics. To mitigate potential imbalances in baseline characteristics (ie, for the comparison in the cost analysis between populations with and without HZ/PHN), propensity score matching (PSM) was applied using a logistic regression model to explore the association between HCRU and costs for patients with HZ/PHN. The model considered baseline confounding variables, and patients were matched on propensity score quintiles. Key variables included were year of index date, age, gender, immunosuppressive conditions, oncology, and presence of chronic conditions.

Direct costs for National Health System in euros (€), derived from all health care resource consumption variables, were extracted. For drug treatments, price at the time of purchase was considered. On the other hand, hospitalizations and outpatient specialist visit/test costs were derived from diagnosis-related group codes and regional tariffs, respectively. Costs for direct health care resources used were reported as average per patient per year. All costs reported—HZ-related only and those that occurred during the follow-up period—were adjusted for the inflation rate reported in Italy and estimated in 2022 €. Finally, an analysis was performed for patients with HZ also considering non-HZ-related costs during the follow-up period.

## RESULTS

### Demographic and Clinical Characteristics

From a total of 193 259 HZ patients identified, 145 923 are ≥50 years. Of these patients, 97.5% were included from outpatient settings, identified by HZ treatment. Demographic and clinical characteristics of these patients are presented in Table 1. The mean age at the index date was 69.6 years, and the Charlson Comorbidity Index score was 0.6. Almost 99.7% of the patients were newly identified cases of HZ, with a higher proportion of females (60.9%). The trend was similar across age groups. The proportion of IC patients (23.5%–34.8%) and those with chronic conditions (50.8%–91.8%) varied among subgroups and increased with age. Details of specific conditions (IC and chronic conditions) across age groups are presented in Supplementary Table 3.

**Table 1.** Demographic and Clinical Characteristics of HZ/PHN Patients With HZ/PHN Groups

	50–59 Years	60–64 Years	65–69 Years	70–79 Years	80+ Years	Total
No.	32 867	19 689	21 452	39 700	32 215	145 923
Age, mean (SD), y	54.8 (2.8)	62.0 (1.4)	67.0 (1.4)	74.4 (2.9)	85.3 (4.3)	69.6 (11.3)
Male, No. (%)	12 270 (37.3)	7861 (39.9)	9236 (43.1)	16 607 (41.8)	11 118 (34.5)	57 092 (39.1)
CCI, mean (SD)	0.3 (0.7)	0.4 (0.8)	0.5 (0.9)	0.7 (1.0)	0.8 (1.0)	0.6 (0.9)
CCI, median (IQR)	0 (0–0)	0 (0–1)	0 (0–1)	0 (0–1)	1 (0–1)	0 (0–1)
HZ new cases, No.^a^ (%)	32 769 (99.7)	19 642 (99.8)	21 393 (99.7)	39 571 (99.7)	32 098 (99.6)	145 473 (99.7)
Recurrent HZ, No.^b^ (%)	1130 (3.4)	626 (3.2)	726 (3.4)	1499 (3.8)	1278 (4.0)	5259 (3.6)
PHN new cases, No.^c^ (%)	9 (0.0)	7 (0.0)	13 (0.1)	31 (0.1)	51 (0.2)	111 (0.1)
IC, No.^d^ (%)	7736 (23.5)	5034 (25.6)	6489 (30.2)	13 140 (33.1)	11 208 (34.8)	43 607 (29.9)
Chronic conditions, No.^e^ (%)	16 709 (50.8)	13 303 (67.6)	16 819 (78.4)	34 589 (87.1)	29 558 (91.8)	110 978 (76.1)

Abbreviations: CCI, Charlson comorbidity index; HZ, herpes zoster; IC, immunocompromised; IQR, interquartile range; PHN, post-herpetic neuralgia.

^a^Patients with HZ without PHN and not diagnosed with HZ in the year before their index date.

^b^Subsequent HZ diagnosis identified during the follow-up period.

^c^Patients with PHN with or without HZ and not diagnosed with PHN in the year before their index date.

^d^Presence of at least 1 treatment.

^e^Presence of at least 1 condition.

### Frequent Prescriptions, Medications, and Hospitalizations

A total of 98.4% of patients with HZ were prescribed antivirals for systemic use during the follow-up period, with similar proportions across age groups (97.0%–99.3%). The most common medications in this category were brivudine (95.9%–98.8%) and acyclovir (3.8%–4.5%). In addition, antibacterials for systemic use, drugs for acid-related disorders, and agents acting on the renin–angiotensin system were the most frequent prescriptions during the characterization and follow-up periods. However, during these periods, a relatively higher proportion of patients with HZ were prescribed antibacterials for systemic use (64.4%–63.3%) than were those without HZ (49.3%–47.5%) across age subgroups (Supplementary Table 4).

For patients with HZ/PHN, during both the characterization and follow-up periods, hospitalizations related to circulatory (2.9% and 3.2%) and musculoskeletal and connective tissue system (2.2% for both periods) issues were the most frequent. The proportion of hospitalizations in patients with HZ/PHN varied among subgroups and increased with age. Details of the hospitalizations are provided in Supplementary Table 5.

### Progression of Patients to PHN or Non-PHN Complications

**Figure 1.**
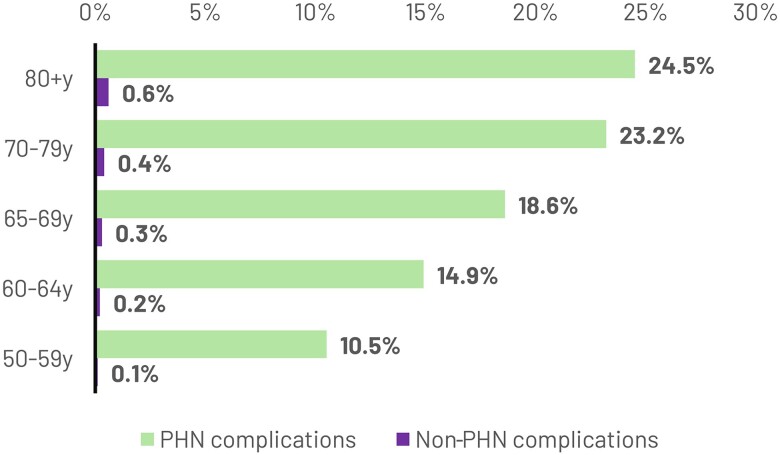
Proportion of patients with HZ with PHN and non-PHN complications. Abbreviations: HZ, herpes zoster; PHN, post-herpetic neuralgia; y, years.

During the follow-up period, 18.8% and 0.35% of patients progressed to PHN and non-PHN complications (neurological [0.10%], ocular [0.05%], other [0.03%], and noncomplicated HZ [0.16%]), respectively. The proportion of patients progressing to PHN or any non-PHN complication increased with age (Figure 1). The proportion of patients with PHN complications was >2 times higher and the proportion of patients with non-PHN complications was >6 times higher in patients aged 80+ years than in patients aged 50–59 years.

### Progression of Outpatients to Inpatient Setting

Of the total number of patients enrolled in an outpatient setting, 448 patients (HZ hospitalizations: 432 [0.3%]; PHN hospitalizations: 16 [0.01%]) progressed to an inpatient setting. This observed phenomenon increased with age, from 36 (0.1%) for patients aged 50–59 years to 180 (0.6%) for patients aged 80+ years.

### Average Length of Stay and Hospitalization

A total of 2.5% (n = 3618) of hospital admissions for HZ were reported, with a median length of stay of 9 days (ordinary hospital admissions: 91.7%; day hospital: 8.3%). The number of hospital admissions increased with age, except for in the 60–64-years age group (Supplementary Figure 2). The highest number of hospitalizations was recorded in the subgroups 70–79 years (n = 1146) and 80+ years (n = 1361).

### Average Direct Health Care Costs of Complications

The average cost of hospital stay ranged from €2813 to €4926. The most frequent hospitalizations were for complications related to the nervous system and to the eyes (Table 2).

**Table 2.** Direct Health Care Costs (€) of Overall Hospital Stay and Type of Hospitalization for Patients With HZ Without PHN

	Patients With at Least 1 Hospitalization, No.	Mean Costs/Hospitalized Patients, €^a^	Total Hospitalizations Costs, €^a^
HZ Without PHN	778	3704	2 882 044
HZ with meningitis	11	4796	52 758
HZ with nervous system complications not specified	29	3583	103 908
HZ of the geniculate ganglion	5	2813	14 063
HZ myelitis	5	4926	24 631
HZ with other complications of the nervous system	56	3572	200 052
Dermatitis of the eyelids from HZ	20	3732	74 631
Keratoconjunctivitis from HZ	46	3667	168 695
Iridocyclitis by HZ	<4	NA	NA
HZ with other ophthalmic complications	26	3524	91 636
External otitis from HZ	13	3321	43 167
HZ with other specified complications	64	3671	234 967
HZ with unspecified complications	38	4038	153 435
HZ without mention of complications	483	3631	1 753 544

Abbreviations: HZ, herpes zoster; NA, not available; PHN, post-herpetic neuralgia.

^a^Adjusted for consumer price index.

### Health Care Resource Consumption

**Figure 2.**
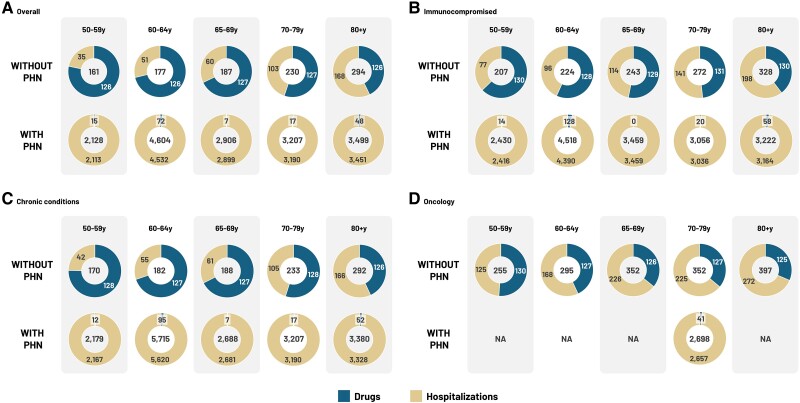
Mean HZ-only cost (€) of HCRU of patients with HZ by age group and PHN status. Abbreviations: HCRU, health care resource utilization; HZ, herpes zoster; NA, not available; PHN, post-herpetic neuralgia; y, years.

The mean HZ-related cost per patient during the first year of follow-up was €217 (drugs: €127; HZ hospitalizations: €91). The total HZ-only HCRU cost for all patients was €31 million, of which costs of drugs related only to HZ (€18 million) were the primary driver, followed by HZ hospitalizations (€13 million). In HZ-only patients (those without PHN), across categories the cost increased with age. While the cost was comparable between the overall (€161–294) and chronic (€170–293) patient groups, it was higher for IC (€208–327) and oncology (€255–397) patients. On the other hand, the use of all categories of health care resources during the first year of follow-up following the index date was higher in patients with HZ with PHN than in patients without PHN. Average health care costs were up to ∼15×, ∼12×, ∼15×, and ∼8× higher for overall, IC, chronic conditions, and oncology patients with HZ with PHN, respectively, compared with those without PHN (Figure 2).

### Direct Cost of Drugs/Prescriptions

Figure 2 presents the cost across age groups and categories. In the overall, IC, chronic conditions, and oncology groups with HZ but without PHN, the average cost of HZ drugs was €126, €130, €127, and €127, respectively. In these patients, the average cost of HZ drugs attributed to the management of patients with HZ tends to decrease with the presence of PHN (€34, €40, €36, and €41). However, the cost of other drugs increased in patients with HZ compared with those without HZ/PHN across categories due to exacerbations (Table 3). The cost of exacerbation (ie, difference in cost of other drugs in the HZ and non-HZ/PHN groups) in the overall, IC, chronic conditions, and oncology groups was €139, €100, €115, and €52, respectively.

**Table 3.** Mean HCRU Cost (€) and Health Care Resource Consumption Post-PSM of Patients With and Without HZ With Underlying Conditions During the First Year of Follow-up

	Immunocompromised		Chronic Conditions		Oncology		Overall	
	Non-HZ/PHN	HZ*	Non-HZ/PHN	HZ*	Non-HZ/PHN	HZ*	Non-HZ/PHN	HZ*
No.	50 161	50 157	119 972	119 823	10 882	10 904	191 308	190 753
HCRU cost, €								
All drugs	951	1180	813	1055	1149	1328	511	777
HZ-related drugs	0	130	0	127	0	127	0	126
Other drugs	951	1051	813	928	1149	1201**	511	650
All-cause hospitalizations	719	877	618	737	1113	1337	395	563
HZ hospitalizations	0	130	0	97	0	219	0	76
Other hospitalizations	719	747***	618	640**	1113	1119***	395**	487
All outpatient service	369	384**	287	311	625	620***	193	250
Total	2039	2441	1718	2103	2887	3285	1099	1590
Health care resource consumption, mean (SD)*								
No.	51 687	51 687	121 984	121 984	11 619	11 619	193 259	193 259
Drug prescriptions	16.3 (13.1)	19.7 (14.5)	16 (11.6)	19.3 (12.7)	17 (13.4)	20 (14.7)	10.1 (11.6)	14.2 (12.6)
Hospitalizations	0.3 (0.7)	0.3 (0.9)	0.2 (0.6)	0.3 (0.7)	0.4 (1.0)	0.5 (1.2)	0.1 (0.5)	0.2 (0.6)
Outpatient services	4.7 (7.2)	5.1 (7.3)	3.9 (5.5)	4.3 (6.0)	7.3 (8.6)	7.5 (9.0)	2.6 (4.8)	3.4 (5.4)

Abbreviations: HCRU, health care resource utilization; HZ, herpes zoster; PHN, post-herpetic neuralgia.

*P < .001, **P < .05, ***P > .05.

### Direct Cost of Hospitalizations

In patients with HZ but without PHN, the cost of HZ hospitalizations increased with age across groups. For patients with HZ and with cancer but without PHN, the costs (€215) were higher than for those in the IC (€135), chronic conditions (€99), and overall (€89) groups. However, in chronic conditions patients with HZ and chronic conditions with PHN, the costs of HZ hospitalizations (€3276) were greater than for those in the IC (€3171), overall (€3284), and oncology (€2657) groups. The cost of hospitalizations for oncology patients with PHN can be considered an outlier as it includes values from the 70–79-years age group only (Figure 2). The cost of exacerbation in the overall, IC, chronic conditions, and oncology groups was €92, €28, €22, and €6, respectively.

### Direct Cost of Outpatient Services

The cost of outpatient services was considerably greater in the IC, chronic conditions, and cancer groups of patients with HZ with/without PHN compared with overall patients with HZ (Supplementary Figure 3). With a few exceptions, the outpatient services cost increased with age. The cost of outpatient services attributable to HZ was greater in patients with PHN (IC: €764.66; chronic conditions: €559.37; cancer: €1433.13; overall: €520.40) when compared with those without PHN (IC: €405.23; chronic conditions: €309.75; cancer: €628.03; overall: €285.17). The cost of outpatient services was almost twice that for those without PHN. However, data were not available for costs of outpatient services associated with HZ only.

### Non-HZ-Related Direct Costs

Regarding non-HZ-related costs for a patient diagnosed with HZ, including drugs, hospitalizations, and outpatient services, the mean cost per patient with HZ during the first year of follow-up was €1896 (drugs: €925; all-cause hospitalizations: €680; and outpatient services: €290). The total HCRU cost thus accounted for €272 million; drug costs (€133 million) were the primary driver, followed by hospitalizations (€98 million) and outpatient services (€42 million). Supplementary Figure 3 presents the total HCRU costs (HZ- and non-HZ-related) across categories for patients with and without PHN.

### Health Care Resource Utilization Post-PSM

Baseline demographic and clinical characteristics of patients with and without HZ/PHN before and after PSM are presented in Supplementary Table 6. After PSM, 193 259 patients with and without HZ/PHN were included (IC: 51 687; chronic conditions: 121 984; oncology: 11 619). The groups were balanced, as evidenced by standardized mean differences of <0.2. In these matched populations, the overall mean age was ∼61.5 years (IC: ∼66 years; chronic conditions: 69 years; oncology: 69 years), with males accounting for ∼40.5% (IC: ∼38%; chronic conditions: 39.5%; oncology: 40%). Similarly, the groups stratified by comorbidities were also well balanced.

Post-PSM, the mean HCRU cost was significantly (*P* < .001) higher for patients with HZ/PHN compared with patients without HZ/PHN (Table 3). An additional increase in costs of €288 in total for the overall group, more specifically for other drugs (€139), other hospitalizations (€92), and outpatient service (€57), contributed greatly to these differences. This was driven by exacerbations due to HZ/PHN, as also evident by a significant (*P* < .001) increase in health care resource consumption for patients with HZ/PHN in the form of drug prescriptions, hospitalizations, and outpatient services compared with patients without HZ/PHN. Similarly, the mean HCRU cost and health care resource consumption was significantly (*P* < .001) higher for the IC, chronic conditions, and oncology patients with HZ/PHN compared with patients without HZ/PHN in these categories, with a few exceptions. The difference in cost between HZ and non-HZ/PHN as a result of exacerbation in the IC, chronic conditions, and oncology groups was €143, €161, and €53, respectively.

## DISCUSSION

To the best of our knowledge, this retrospective study represents the largest and most recent update of HCRU and direct costs attributed to management of HZ or/and PHN in older adult patients in Italy, stratified by age group and underlying conditions. Among individuals aged ≥50 years, we found that direct HCRU and costs linked to HZ show an upward trend as individuals get older, especially in those with chronic illnesses, IC, and cancer, in particular when complicated by PHN. Costs are also significantly higher when compared with those without HZ post-PSM. The higher direct HCRU costs among patients with HZ/PHN were driven by greater resource consumption in the form of drug prescriptions, hospitalizations, and outpatient services.

Patients with HZ/PHN aged ≥50 years impose a significant economic burden on the Italian population, with total direct health care costs exceeding €272 million per year. These estimates are representative of the patients who were included in the study and include costs associated with other related diseases as well. Of these, the total HZ-only cost for all patients was €31.21 million. The estimates observed in our study are comparable to those reported by Gialloreti et al., who reported costs of €28.2 million in similar settings, not including IC patients [16]. In another study from the United States, HZ and its complications were projected to incur direct medical costs of $2.4 billion in those aged ≥18 years, with a likelihood of 1.1 million HZ cases annually [17]. Furthermore, in our study, the average direct health care cost per patient with a primary diagnosis of HZ was higher for IC, chronic conditions, and cancer compared with the overall HZ/PHN group (€1896). The costs of HZ-only prescriptions and hospitalizations were €217. However, the costs of outpatient visits due to HZ only could not be extracted in this study. Several studies conducted in different locations and patient groups quote the mean direct health care cost per patient. In the United States, the mean cost per patient at 1 year was US$1052–3815 for all HZ cases [18]. In Argentina, Brazil, and Mexico, the direct total costs per patient ranged from US$1125 to $4177.91 [19]. In patients in Italy with chronic conditions, for example, COPD, the mean direct cost per patient was €2461 [20], and for IC patients in the United States (eg, those with HIV, cancer), costs ranged from $2549 to $3108 in the first quarter post–index date [21]. In a US study by White et al., the direct medical costs for IC patients were almost 2 times greater compared with those who were not IC [18]. In patients with psoriasis, COPD, ulcerative colitis, Crohn's disease, and rheumatoid arthritis with HZ, the total health care costs were $5020, $6278, $6515, $9910, and $7070 at 1 month, driven mainly by higher inpatient costs [22–25]. However, it is difficult to compare these studies because of differences in study design, demographics, location, time period, pricing, socioeconomic structure, etc. Nonetheless, all these studies including the present one show the high cost of managing patients with HZ/PHN, and those with comorbid conditions put a significant burden on health care systems.

In relation to health care resource use, we found that medication accounted for a significant portion (48.81%) of the total health care cost (€272 million), followed by hospitalization and outpatient services. This is equivalent to what Matthews et al. [26]. reported (ie, ∼49%) in patients in Italy aged 50 years or older [26]. Antivirals were the most frequent medication used in HZ treatment [1]. In our study total, 98.4% of patients were prescribed antivirals, especially brivudine and acyclovir. The expensiveness of these drugs threatens health care budgets and limits funds that could be used for other areas of public interest [26, 27]. Also, despite these drugs being effective, they need to be given within 72 hours to produce an adequate response, which is often not the case because of delays in seeking medical help. Even timely intervention is unable to prevent patients from progressing to PHN, one of the most common and debilitating sequelae of HZ [28]. Further hospitalization is of concern among the elderly as it is the result of serious manifestations of HZ disease. The present study reported a median length of stay of 9 days due to hospitalizations, with the highest number of hospitalizations occurring in those aged >70 years. These findings are similar to the findings of Scampoli et al. [29] in Italy, who also reported a median length of stay of 9 days and increased hospitalizations with age (60–74 years: 26.1%; >74 years: 37.6%). The average cost of hospital stay ranged from €2813 to €4926, which was comparable to the cost of hospital admission (€2695) reported by Matthews et al. [26]. The slightly higher cost observed in our study could be the result of increased treatment cost over time and also because of inclusion of patients with various comorbidities.

In the present study, 18.8% of the patients progressed to PHN complications, a proportion that increased with age to more than double for patients aged 80+ years compared with the 50–59-years group. These rates are consistent with those reported in various other community-based studies (17.4%–22.7%) [30–32]. Management of patients with HZ with complications is resource-intensive, inflicting a large burden on the health care system. When stratified by age group and underlying conditions, the results showed that the direct HCRU costs over the 1-year follow-up period increased with age and were significantly higher in patients with HZ with PHN (up to 4 times overall and up to 15 times for HZ-only) compared with those without PHN. The higher costs in patients with PHN could be partially related to the fact that PHN was defined based on the ICD-9 code, while patients without PHN comprise both inpatient and outpatient individuals, without a PHN-specific code at the index date. Across the age groups and in those with underlying conditions, while medication costs account for the main proportion of total costs in patients with HZ but without PHN, hospitalization costs are the major contributor for patients with PHN. These results are in line with previous findings wherein costs of prescription drugs and hospitalization were the main drivers for patients with HZ and PHN, respectively [33, 34].

Limitations of this study are the following. First, as this was a retrospective study of records from large databases, inaccuracies cannot be excluded; for example, we could extract overall outpatient visits and not outpatient visits due only to HZ. Second, persons who do not seek medical advice or refuse to be treated might lead to an underestimation of the proportion of HZ/PHN cases. Third, as the costs presented are HZ-related only during the follow-up period, we do not know specifically whether all of these are linked with HZ exacerbation. The exacerbation cost in patients with HZ with comorbid conditions, such as compromised immune systems or chronic conditions, was less compared with that in patients overall, which could be due to the fact that patients with comordibities already have additional HCRU costs because of their underlying conditions. However, to our knowledge, this is the most recent study to evaluate the direct HCRU and costs of patients with HZ/PHN in Italy, with a large sample size. Results showed high medical costs related to medications, hospitalizations, and outpatient visits among older adults and individuals with comorbidities, particularly when complicated by PHN. Because HZ and PHN episodes represent a significant economic burden to the health care system, identification of cost-effective strategies is important. Data from this study provide valuable information to clinicians and could help policy-makers to refine strategies, such as managing HZ through prevention (eg, vaccination).

## Supplementary Material

ofae738_Supplementary_Data

## Supplementary Data

Supplementary materials are available at *Open Forum Infectious Diseases* online. Consisting of data provided by the authors to benefit the reader, the posted materials are not copyedited and are the sole responsibility of the authors, so questions or comments should be addressed to the corresponding author.
