# Supplementary material for: The Economic Burden of Herpes Zoster in Individuals Aged 50 Years or Older and Those With Underlying Conditions in Italy
Source: Open Forum Infect Dis. 2024 Dec 20;12(1):ofae738. doi: 10.1093/ofid/ofae738 (PMC11773188; doi:10.1093/ofid/ofae738)
Supplement: ofae738_Supplementary_Data [file ofae738_supplementary_data.docx]

**Supplementary Material**

**Methods: Patients and Data Source**

All patients (≥18 years) with at least one record of herpes zoster (HZ) and/or postherpetic neuralgia (PHN) hospitalization discharge diagnosis (i.e. inpatient) or a prescription of brivudine (ATC [Anatomical Therapeutic Chemical] code: J05AB15) for HZ (i.e. outpatient) with 12 months of data before and after discharge or brivudine prescription were included in the study. They were stratified according to age group (50–59, 60–64, 65–69, 70–79 and ≥80 years), HZ (PHN and non-PHN complications), immunocompromised (IC) conditions, chronic conditions, and oncology. Patients with PHN complications were identified by the prescription of pregabalin, gabapentin and tramadol for one year of follow-up and/or by the presence of discharge diagnosis for hospitalization using the ATC and International Classification of Diseases, Ninth Revision, Clinical Modification (ICD-9-CM) codes (Supplementary Tables 1 and 2). A control group of patients without HZ/PHN was identified after propensity score matching (PSM) and balanced with the relative HZ/PHN population for the demographic and clinical characteristics. Patients transferred to a different Local Health Authority (LHA) during the study period were excluded.

### *Data sources*: In Italy, the National Health Service (NHS) provides all residents a wide range of healthcare services. The following databases were used to obtain information related to patients enrolled in this study:

- Beneficiaries database: Patient demographics;
- Pharmaceuticals database: Drug supplies, prescription date, drug package ID, number of packages and costs per package;
- Hospitalization database: Hospitalizations (e.g., admission and discharge date, type of hospitalization, main and secondary diagnosis, status of discharged patients, main and secondary procedures, diagnosis-related group);
- Diagnostic tests and specialist visits database: Referral information, including diagnostic tests and visits
- Pathology database: Supplied exemption ticket, including information on exemption type.

Each patient was assigned an anonymous numeric code to keep privacy and allow electronic linking between databases. The results of the analysis were produced in aggregated form so that it would be impossible to attribute them to a single institution, doctor, individual or individual prescribing behaviors directly or indirectly. Data from each LHA were anonymized in compliance with Italian code (protection of personal data: Legislative Decree 196/2003, as amended by 101/2018) and European Union General Data Protection Regulation 679/2016 provisions.

As this was a retrospective observational study, informed consent was not required. The local ethics committee was notified of the study, which was conducted in accordance with the ethical principles from the Declaration of Helsinki, also, consistent with Good Pharmacoepidemiology Practices, and the applicable laws, and regulations of the country.

# Supplementary Table S1. ICD-9-CM Codes for Hospital Diagnosis of Herpes Zoster

| **ICD-9-CM code** | **Label** |
| --- | --- |
| 053.0 | Herpes zoster with meningitis |
| 053.10 | Herpes zoster with nervous system complications not specified |
| 053.11 | Herpes zoster del ganglio geniculate |
| 053.14 | Herpes zoster myelitis |
| 053.19 | Herpes zoster with other complications of the nervous system |
| 053.20 | Dermatitis of the eyelids from herpes zoster |
| 053.21 | Keratoconjunctivitis from herpes zoster |
| 053.22 | Iridocyclite by herpes zoster |
| 053.29 | Herpes zoster with other ophthalmic complications |
| 053.71 | External otitis from herpes zoster |
| 053.79 | Herpes zoster with other specified complications |
| 053.8 | Herpes zoster with not specified complications |
| 053.9 | Herpes zoster without complications |
| 053.12 | Postherpetic trigeminal neuralgia |
| 41.0 | Haematopoietic stem cell transplantation |
| V42.X | Organ transplantation |
| 140–239, ATC L01, exemption 048 | Tumors |
| 714 or exemption code: 006 | Rheumatoid arthritis |
| 710.0 and/or an active exemption code 028.710.0 | Systemic lupus erythematosus |
| 555, 556 or exemption code:  009.555, 009.556 | Inflammatory bowel disease |
| 696.1, or exemption code 045.696.1 | Psoriasis or by at least 1 prescription for antipsoriatic topical drugs (ATC code: D05AA) |
| 042 | Human immunodeficiency virus |
| V42.0, V56.0, or 55.6, 39.95, 54.98, or 39.95, 54.98 | End-stage renal disease |
| 430–438, 428, 410, 411, 413, 414,427.31 | Cardiovascular events including stroke, heart failure, acute myocardial infarction, atrial fibrillation |
| 00.4X, 00.5X, 00.6X, 35.XX-39.XX | Cardiovascular procedures |

ICD-9-CM, International Classification of Diseases, Ninth Revision, Clinical Modification.

# Supplementary Table S2. Anatomical Therapeutic Chemical Codes

| N03AX16 | Pregabalin |
| --- | --- |
| N03AX12 | Gabapentin |
| N02AX02 | Tramadol |
| L04, L03AB, L03AX13, N07XX09, H02 | Immunosuppressive therapy identified by the presence of at least one prescription of immunosuppressant, interferons, glatiramer acetate, dimethylfumarate or systemic corticosteroids |
| A10 | Diabetes |
| C10 | Dyslipidemia |
| C02, C03, C07, C08, C09 | Hypertension |

# Supplementary Table S3. Clinical Characteristics of HZ Patients During the Characterization Period

|  | **50–59 years** | **60–64 years** | **65–69**  **years** | **70–79**  **years** | **80+**  **years** | **Total** |
| --- | --- | --- | --- | --- | --- | --- |
| N | 32,867 | 19,689 | 21,452 | 39,700 | 32,215 | **1,45,923** |
| **Immunocompromised patients, N(%)** | | | | | | |
| HSCT | 8 (0.0) | 4 (0.0) | 8 (0.0) | NI | 0 (0.0) | 23 (0.0) |
| Organ transplantation | 23 (0.1) | 26 (0.1) | 26 (0.1) | 33 (0.1) | 18 (0.1) | 126 (0.1) |
| Tumors | 1,583 (4.8) | 1,204 (6.1) | 1,674 (7.8) | 3,598 (9.1) | 2,511 (7.8) | 10,570 (7.2) |
| RA | 162 (0.5) | 120 (0.6) | 157 (0.7) | 217 (0.5) | 127 (0.4) | 783 (0.5) |
| SLE | 20 (0.1) | 6 (0.0) | 11 (0.1) | 12 (0.0) | NI | 51 (0.0) |
| IBD | 61 (0.2) | 38 (0.2) | 33 (0.2) | 72 (0.2) | 39 (0.1) | 243 (0.2) |
| Psoriasis | 31 (0.1) | 14 (0.1) | 13 (0.1) | 20 (0.1) | 9 (0.0) | 87 (0.1) |
| HIV | 15 (0.0) | 5 (0.0) | NI | NI | 0 (0.0) | 25 (0.0) |
| ESRD | 38 (0.1) | 38 (0.2) | 52 (0.2) | 114 (0.3) | 79 (0.2) | 321 (0.2) |
| IT | 6,627 (20.2) | 4,206 (21.4) | 5,386 (25.1) | 10,828 (27.3) | 9,541 (29.6) | 36,588 (25.1) |
| **Chronic condition patients, N(%)** | | | | | | |
| Diabetes | 2,442 (7.4) | 2,679 (13.6) | 3,763 (17.5) | 8,616 (21.7) | 6,774 (21.0) | 24,274 (16.6) |
| Dyslipidemia | 5,362 (16.3) | 5,588 (28.4) | 7,973 (37.2) | 17,663 (44.5) | 12,182 (37.8) | 48,768 (33.4) |
| Hypertension | 11,872 (36.1) | 10,432 (53.0) | 14,090 (65.7) | 30,900 (77.8) | 27,732 (86.1) | 95,026 (65.1) |
| COPD | 5,504 (16.7) | 3,765 (19.1) | 5,061 (23.6) | 10,728 (27.0) | 9,975 (31.0) | 35,033 (24.0) |
| Cardiovascular events | 409 (1.2) | 441 (2.2) | 714 (3.3) | 2,099 (5.3) | 2,853 (8.9) | 6,516 (4.5) |
| Stroke | 98 (0.3) | 109 (0.6) | 190 (0.9) | 636 (1.6) | 1,026 (3.2) | 2,059 (1.4) |
| Heart failure | 46 (0.1) | 59 (0.3) | 119 (0.6) | 477 (1.2) | 885 (2.7) | 1,586 (1.1) |
| AMI | 149 (0.5) | 190 (1.0) | 312 (1.5) | 787 (2.0) | 825 (2.6) | 2,263 (1.6) |
| Atrial fibrillation | 47 (0.1) | 67 (0.3) | 149 (0.7) | 543 (1.4) | 944 (2.9) | 1,750 (1.2) |
| Cardiovascular procedures | 228 (0.7) | 216 (1.1) | 299 (1.4) | 756 (1.9) | 677 (2.1) | 2,176 (1.5) |

AMI, acute myocardial infarction; COPD, chronic obstructive pulmonary disease; ESRD: end-stage renal disease; HSCT, haematopoietic stem cell transplantation; HIV, human immunodeficiency virus; HZ, herpes zoster; IBD, infammatory bowel disease; IT, immunosuppressive therapy; N: number of patients; NI, not indicated; RA, rheumatoid arthritis; SLE, systemic lupus erythematosus.

# Supplementary Table S4. Most Frequent Drug Prescription During Characterization and Follow-up Period

| **ATC code** |  | **Characterization period** | | | | | | **Follow-up period** | | | | | |
| --- | --- | --- | --- | --- | --- | --- | --- | --- | --- | --- | --- | --- | --- |
|  | **Age groups, years** | **50–59** | **60–64** | **65–69** | **70–79** | **80+** | **Total** | **50–59** | **60–64** | **65–69** | **70–79** | **80+** | **Total** |
|  | **Patients without HZ, N** | 30,736 | 18,025 | 20,421 | 38,104 | 34,927 | **142,213** | 30,736 | 18,025 | 20,421 | 38,104 | 34,927 | **142,213** |
| **J01** | Antibacterials for systemic use (%) | 47.3 | 50.7 | 51.9 | 52.4 | 45.5 | **49.3** | 44.6 | 48.8 | 51.0 | 50.7 | 43.8 | **47.5** |
|  | **Age groups, years** | **50–59** | **60–64** | **65–69** | **70–79** | **80+** | **Total** | **50–59** | **60–64** | **65–69** | **70–79** | **80+** | **Total** |
|  | **Patients with HZ, N** | 32,867 | 19,689 | 21,452 | 39,700 | 32,215 | **145,923** | 32,867 | 19,689 | 21,452 | 39,700 | 32,215 | **145,923** |
| **J05** | Antivirals for systemic use (%) | - | - | - | - | - | **-** | 99.3 | 99.1 | 98.9 | 98.1 | 97.0 | **98.4** |
|  | Brivudine (%) | 0.3 | 0.2 | 0.2 | 0.2 | 0.2 | **0.2** | 98.8 | 98.5 | 98.1 | 97.2 | 95.9 | **97.6** |
|  | Acyclovir (%) | 3.1 | 3.0 | 3.0 | 3.4 | 3.3 | **3.2** | 3.8 | 3.9 | 4.5 | 4.4 | 4.3 | **4.2** |
|  | Valacyclovir (%) | 0.7 | 0.8 | 0.7 | 1.0 | 0.9 | **0.9** | 0.9 | 1.0 | 1.3 | 1.3 | 1.3 | **1.1** |
|  | Famciclovir (%) | 0.3 | 0.3 | 0.4 | 0.4 | 0.5 | **0.4** | 0.5 | 0.5 | 0.6 | 0.6 | 0.6 | **0.6** |
|  | Immunosuppressants (%) | 1.7 | 1.7 | 1.9 | 1.5 | 0.8 | **1.5** | 1.7 | 1.8 | 1.9 | 1.4 | 0.9 | **1.5** |
| **J01** | Antibacterials for systemic use (%) | 57.1 | 60.2 | 65.8 | 67.9 | 69.4 | **64.4** | 56.7 | 59.5 | 65.6 | 66.0 | 67.5 | **63.3** |
| **A02** | Drugs for acid related disorders (%) | 36.4 | 44.8 | 56.0 | 65.8 | 72.7 | **56.4** | 38.2 | 47.0 | 58.8 | 67.9 | 74.0 | **58.4** |
| **C09** | Agents acting on the renin-angiotensin system (%) | 27.3 | 42.3 | 53.6 | 64.7 | 69.3 | **52.6** | 29.5 | 44.5 | 55.3 | 65.4 | 66.3 | **53.2** |
| **M01** | Antiinflammatory and antirheumatic products (%) | 38.8 | 44.0 | 52.1 | 55.7 | 64.3 | **49.1** | 40.2 | 45.4 | 53.7 | 55.2 | 50.0 | **49.1** |
| **B01** | Antithrombotic agents (%) | 12.0 | a | 32.7 | a | 64.3 | **37.9** | a | a | a | a | a | **39.7** |
| **C10** | Lipid modifying agents (%) | 16.3 | 28.4 | 37.2 | 44.5 | 37.8 | **33.4** | 17.9 | 30.0 | 38.7 | 45.0 | 36.1 | **34.0** |
| **C07** | Beta blocking agents (%) | 14.6 | 22.1 | 28.0 | 33.6 | 35.6 | **27.4** | 15.9 | 23.5 | 29.6 | 35.2 | 36.9 | **28.8** |
| **H02** | Corticosteroids for systemic use (%) | 19.5 | 20.7 | 24.4 | 26.8 | 29.4 | **24.5** | 20.2 | 21.6 | 25.9 | 27.6 | 30.3 | **25.5** |
| **R03** | Drugs for obstructive airway diseases (%) | 16.7 | 19.1 | 23.6 | 27.0 | 31.0 | **24.0** | 16.7 | a | a | a | a | - |
| **A11** | Vitamins (%) | 14.3 | 18.9 | 22.2 | 26.7 |  | **22.3** | 17.0 | 21.5 | 25.4 | 28.9 | a | **24.6** |

a: not present in 10 most frequently prescribed drug list. HZ, herpes zoster; N, number of patients.

# Supplementary Table S5. Most Frequent Hospitalizations During Characterization and Follow-up Period

|  | **Characterization period** | | | | | | **Follow-up period** | | | | | |
| --- | --- | --- | --- | --- | --- | --- | --- | --- | --- | --- | --- | --- |
| **Age groups, years** | **50–59** | **60–64** | **65–69** | **70–79** | **80+** | **Total** | **50–59** | **60–64** | **65–69** | **70–79** | **80+** | **Total** |
| **N** | 32,867 | 19,689 | 21,452 | 39,700 | 32,215 | **145,923** | 32,867 | 19,689 | 21,452 | 39,700 | 32,215 | **145,923** |
| Circulatory system (%) | 1.1 | 1.8 | 2.5 | 3.6 | 5.1 | **2.9** | 1.1 | 1.9 | 2.3 | 3.9 | 5.8 | **3.2** |
| Musculoskeletal system & connective tissue (%) | 1.4 | 1.9 | 2.1 | 2.5 | 2.8 | **2.2** | 1.6 | 1.7 | 2.2 | 2.5 | 2.7 | **2.2** |
| Respiratory system (%) | a | a | a | a | a | **1.4** | 0.6 | 1.0 | 1.3 | 2.6 | 4.3 | **2.1** |
| Nervous system (%) | 0.5 | 0.8 | 0.9 | 1.5 | 2.4 | **1.3** | 0.8 | 1.1 | 1.3 | 2.4 | 3.6 | **2.0** |
| Digestive system (%) | 0.9 | 1.2 | 1.4 | 1.7 | 1.7 | **1.4** | 0.9 | 1.3 | 1.4 | 1.8 | 2.0 | **1.5** |
| Kidney and urinary tract (%) | 0.4 | 0.6 | 0.8 | 1.1 | 1.4 | **0.9** | 0.5 | 0.7 | 0.8 | 1.3 | 1.8 | **1.1** |
| Skin, subcutaneous tissue & breast (%) | 0.5 | a | 0.6 | 0.7 | 0.8 | **0.6** | 0.7 | 0.8 | 1.0 | 1.2 | 1.4 | **1.0** |
| Myeloproliferative DDs (poorly differentiated neoplasms) (%) | 0.5 | 0.7 | 1.0 | 0.9 | 0.6 | **0.8** | 0.5 | 0.7 | 1.0 | 1.1 | 0.8 | **0.8** |
| Hepatobiliary system & pancreas (%) | 0.5 | 0.6 | 0.8 | 0.8 | 0.8 | **0.7** | 0.5 | 0.7 | 0.8 | 1.0 | 1.0 | **0.8** |
| Eye (%) | a | a | a | a | a | **0.5** | a | a | a | a | a | **0.6** |

a: not present in 10 most frequent hospitalization list. N, number of patients.

# Supplementary Table S6. Demographic and Clinical Characteristics of Study Population Pre and Post PSM

|  | Pre PSM | | Post PSM | | Immunocompromised | | Chronic conditions | | Oncology | |
| --- | --- | --- | --- | --- | --- | --- | --- | --- | --- | --- |
|  | **Non-HZ/PHN N=1,93,259** | **HZ/PHN** | **Non-HZ/PHN** | **HZ/PHN N=193,259** | **Non-HZ/PHN** | **HZ/PHN N=193,259** | **Non-HZ/PHN** | **HZ/PHN N=193,259** | **Non-HZ/PHN** | **HZ/PHN N=193,259** |
| N | 1,511,308 | 193,259 | 193,259 | 193,259 | 51,687 | 51,687 | 121,984 | 121,984 | 11,619 | 11,619 |
| Age, mean (SD) | 51.1 (19.8) | 61.6 (17.8)^a^ | 61.5 (18.7) | 61.6 (17.8)^b^ | 65.8 (16.4) | 66.1 (16.1) | 68.7 (14.7) | 68.9 (14.1) | 68.4 (14.0) | 68.7 (13.2) |
| Male | 730,627 | 78,258 | 78,710 | 78,258^b^ | 19,737 | 19,355 | 48,493 | 48,219^b^ | 4,651 | 4,618^b^ |
| Diabetes | 73,982 | 25,227^a^ | 22,979 | 25,227 | 7,031 | 7,309 | NA | NA | 1,951 | 1,994^b^ |
| Dyslipidemia | 138,557 | 50,267^a^ | 45,288 | 50,267 | 16,060 | 16,534 | NA | NA | 3,700 | 3,782^b^ |
| Hypertension | 306,335 | 99,208^a^ | 96,314 | 99,208 | 32,781 | 33,030^b^ | NA | NA | 7,715 | 7,752^b^ |
| COPD | 132,428 | 41,803^a^ | 34,661 | 41,803 | 18,283 | 19,113^b^ | NA | NA | 3,059 | 3,179^b^ |
| Stroke | 5,368 | 2,096 | 1,692 | 2,096 | 861 | 909^b^ | NA | NA | 244 | 241^b^ |
| Heart failure | 3,570 | 1,600 | 1,222 | 1,600 | 756 | 798^b^ | NA | NA | 172 | 178^b^ |
| AMI | 6,026 | 2,309 | 2,039 | 2,309 | 956 | 988^b^ | NA | NA | 299 | 311^b^ |
| AF | 3,961 | 1,762 | 1,364 | 1,762 | 771 | 838^b^ | NA | NA | 227 | 248^b^ |
| CV procedures | 6,555 | 2,325 | 2,113 | 2,325 | 1,001 | 1,033^b^ | NA | NA | 372 | 366^b^ |
| HSCT | 36 | 38 | 19 | 38 | NA | NA | 14 | 32 | 19 | 37 |
| OT | 368 | 158 | 139 | 158^b^ | NA | NA | 128 | 144^b^ | 33 | 49^b^ |
| Tumors | 34,378 | 11,619 | 9,638 | 11,619 | NA | NA | 8,654 | 9,125 | NA | NA |
| RA | 2,041 | 866 | 601 | 866 | NA | NA | 566 | 682 | 220 | 223^b^ |
| SLE | 288 | 85 | 86 | 85^b^ | NA | NA | 70 | 67^b^ | 13 | 14^b^ |
| IBD | 1,551 | 340 | 345 | 340^b^ | NA | NA | 209 | 221^b^ | 40 | 34^b^ |
| Psoriasis | 454 | 108 | 100 | 108^b^ | NA | NA | 83 | 76^b^ | 14 | 11^b^ |
| HIV | 163 | 64 | 63 | 64^b^ | NA | NA | 28 | 36^b^ | 5 | 9^b^ |
| ESRD | 1,042 | 356 | 305 | 356 | NA | NA | 329 | 340^b^ | 47 | 50^b^ |
| IT | 133,942 | 43,888 | 35,757 | 43,888 | NA | NA | 32,418 | 34,619 | 4,322 | 4,447^b^ |
| CI | 0.2 | 0.5 | 0.4 | 0.5 | 0.8 | 0.8 | 0.7 | 0.7 | 1.3 | 1.3 |

AF, atrial fibrillation; AMI, acute myocardial infarction; CI, Charlson Index; COPD, chronic obstructive pulmonary disease; CV, cardiovascular; ESRD, end-stage renal disease; HIV: human immunodeficiency virus; HSCT, haematopoietic stem-cell transplantation; HZ, herpes zoster; IBD, inflammatory bowel disease; IT, immunosuppressive therapy; NA, not available; OT, organ transplantation; PHN, postherpetic neuralgia; PSM, propensity score matching; RA, rheumatoid arthritis; SD, standard deviation; SLE: systemic lupus erythematosus; SMD, standardized mean difference.

a: SMD >0.2.

b: p >0.05

# Supplementary Figure S1: Study Design


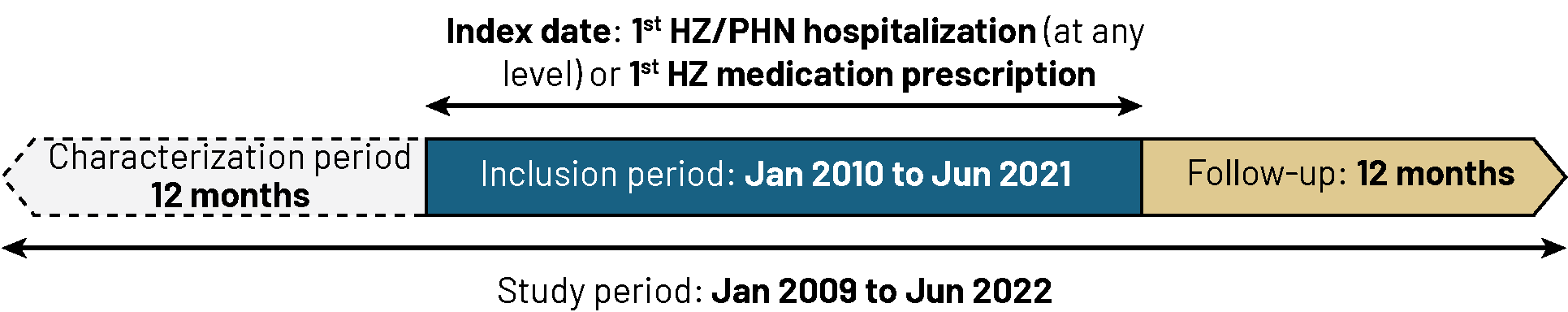


HZ, Herpes zoster; PHN, postherpetic neuralgia; Jan, January; Jun, June.

**Supplementary Figure S2: Hospitalizations Type and Length of Stay in HZ Patients**


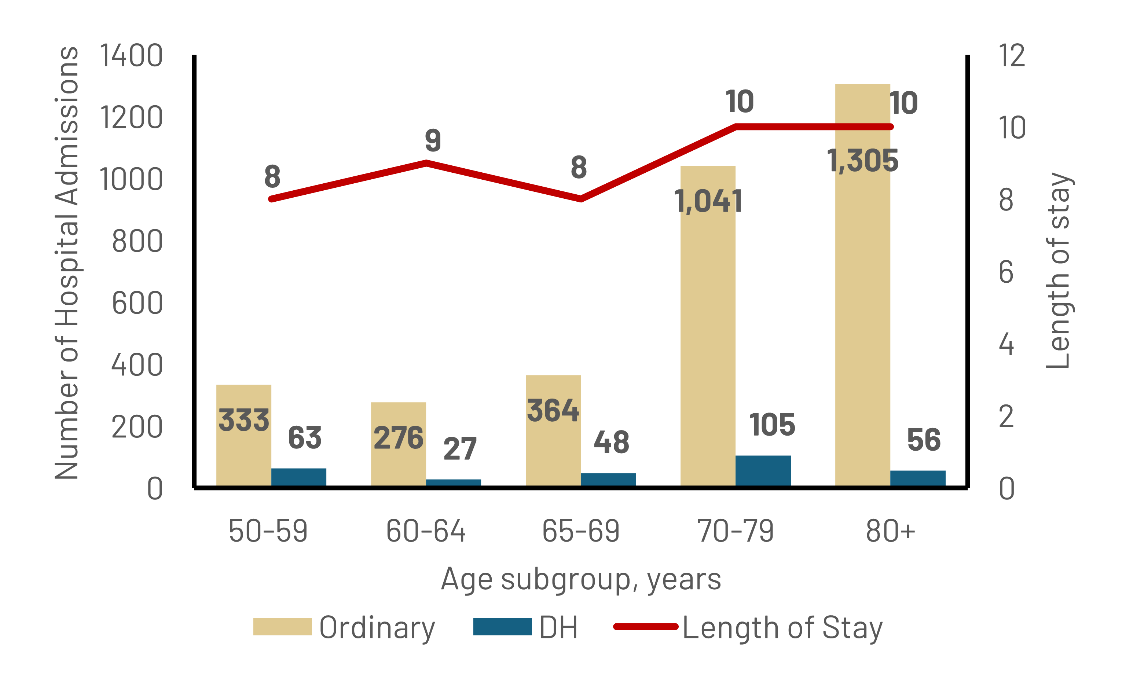


DH, day hospital; HZ, herpes zoster. Length of stay is reported as median.

# Supplementary Figure S3. Mean Cost (€) of HCRU of HZ Patients by Age Group and PHN Status


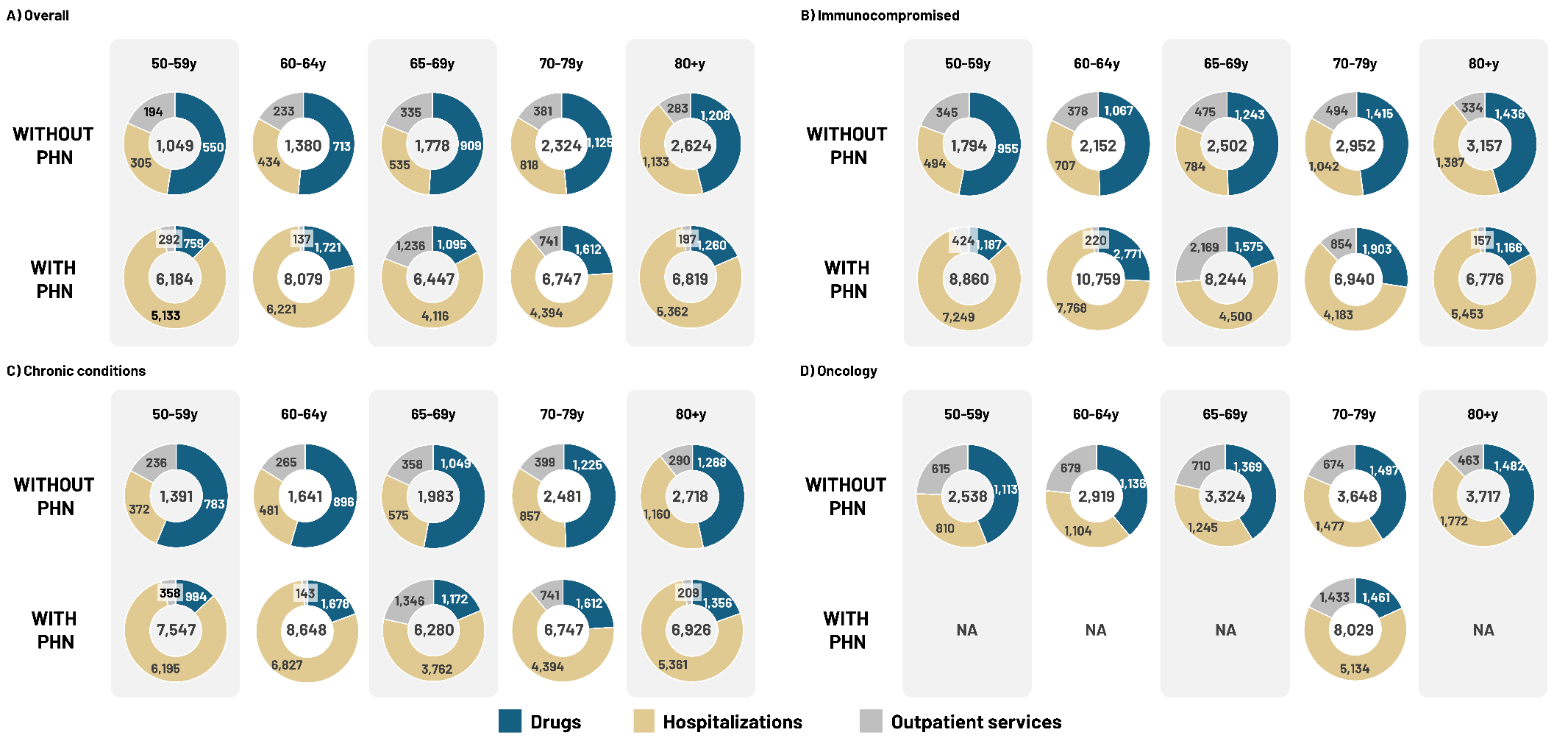


HZ, herpes zoster; PHN, postherpetic neuralgia; HCRU, healthcare resource utilization; NA, not available; y, years.
